# Multiple Cardiac Rhabdomyomas, Wolff-Parkinson-White Syndrome, and Tuberous Sclerosis: An Infrequent Combination

**DOI:** 10.1155/2014/973040

**Published:** 2014-09-22

**Authors:** Elena Castilla Cabanes, Isaac Lacambra Blasco

**Affiliations:** ^1^Echocardiography Section, Department of Cardiology, Hospital Clínico Universitario de Zaragoza, Avenida San Juan Bosco 15, 50009 Zaragoza, Spain; ^2^Department of Cardiology, Hospital General Universitario de Elche, Camí de L'Almassera 11, Alicante, 03023 Elche, Spain

## Abstract

Cardiac rhabdomyomas are benign cardiac tumours and are often associated with tuberous sclerosis. They are often asymptomatic with spontaneus regresion but can cause heart failure, arrhythmias, and obstruction. There have also been a few isolated reports of Wolff-Parkinson-White syndrome occurring in association with tuberous sclerosis and the great majority has been detected in patients with concomitant rhabdomyomas. We report a 12-day-old infant girl with tuberous sclerosis who presented with intraparietal and intracavitary rhabdomyomas with a Wolff-Parkinson-White syndrome (WPW). She represents one of the few published cases of WPW syndrome and tuberous sclerosis and particularly interesting because of intramural rhabdomyomas regression with persistent intracavitary rhabdomyomas after two years of followup.

## 1. Introduction

Cardiac rhabdomyomas are benign cardiac tumours and are often associated with tuberous sclerosis [1]. They must be investigated by echocardiography in this setting. They are often asymptomatic but can cause heart failure, arrhythmias, and obstruction. In these cases, they must be operated upon. In other cases—and because of their tendency to regress spontaneously—these tumours are simply monitored by echocardiography and Holter recording, in addition to usual clinical examinations. There have also been a few isolated reports of Wolff-Parkinson-White syndrome occurring in association with tuberous sclerosis and the great majority has been detected in patients with concomitant rhabdomyomas; however its prevalence in this syndrome is unknown.

## 2. Case Report

**Figure 1:**
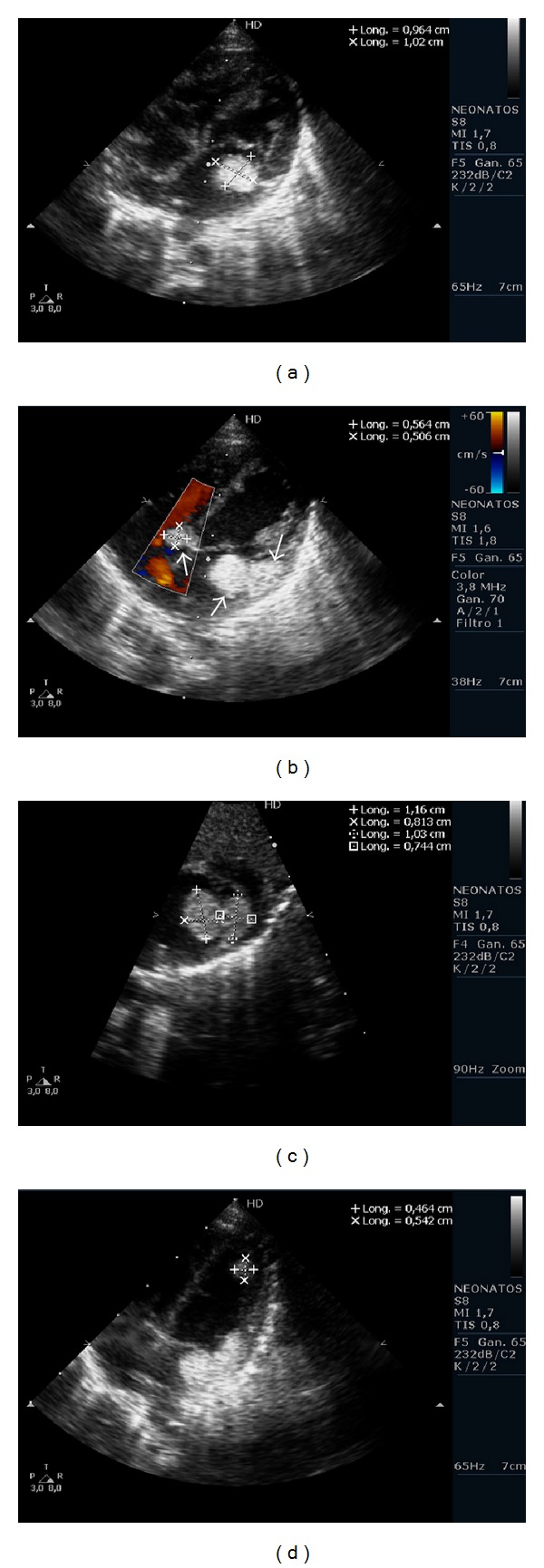
Views of different sizes and localisations of rhabdomyomas at the time of diagnosis. (a) Four-chamber view with intracavitary masses in mitral valve of 0,9 × 1 cm. (b) Four-chamber doppler-colour view of intracavitary mass of 0,5 × 0,5 cm in tricuspid valve, one intracavitary mass in mitral valve and one intraparietal mass in lateral wall of left atrium (arrows). (c) Subcostal view of left ventricule. One intracavitary mass and one intraparietal mass in lateral face of left ventricule. (d) Four-chamber view of intraparietal tumour in left ventricule apex.

We report a 12-day-old infant girl who presented with a supraventricular tachycardia at a rate of 250 beats/min and tachypnea. She was admitted to hospital and electrocardiogram showed supraventricular tachycardia with delta wave and diagnosis of Wolff-Parkinson-White (WPW) syndrome was made. Physical and radiological examination showed pulmonary congestion. She was treated with 0,07 mg/6 hours of intravenous propranolol and 6 mg/day of intravenous furosemide and had a satisfactory evolution. A transthoracic echocardiography was made to look for a possible structural cardiopathy that showed multiple rounded, homogeneous, and echo-dense intracavitary masses in interventricular septum, free lateral wall of left ventricle, free wall of right atrium, mitral valve, and septal tricuspid leaflet that conditioned moderate tricuspid regurgitation; all fit in with multiple congenital cardiac rhabdomyomas (Figure 1). No neurological deficit was detected at the time of diagnosis.

Therefore tuberous sclerosis was thought to be a possible concomitant disease with both WPW syndrome and cardiac rhabdomyomas. A second stigma of tuberous sclerosis was found in cranial magnetic resonance, a subependymal tumour, that allowed achieving the final diagnosis. Thoracic-abdominal-pelvic computed tomography and electroencephalogram were normal. Molecular genetic test for tuberous sclerosis showed mutation of* TSC1* gen.

**Figure 2:**
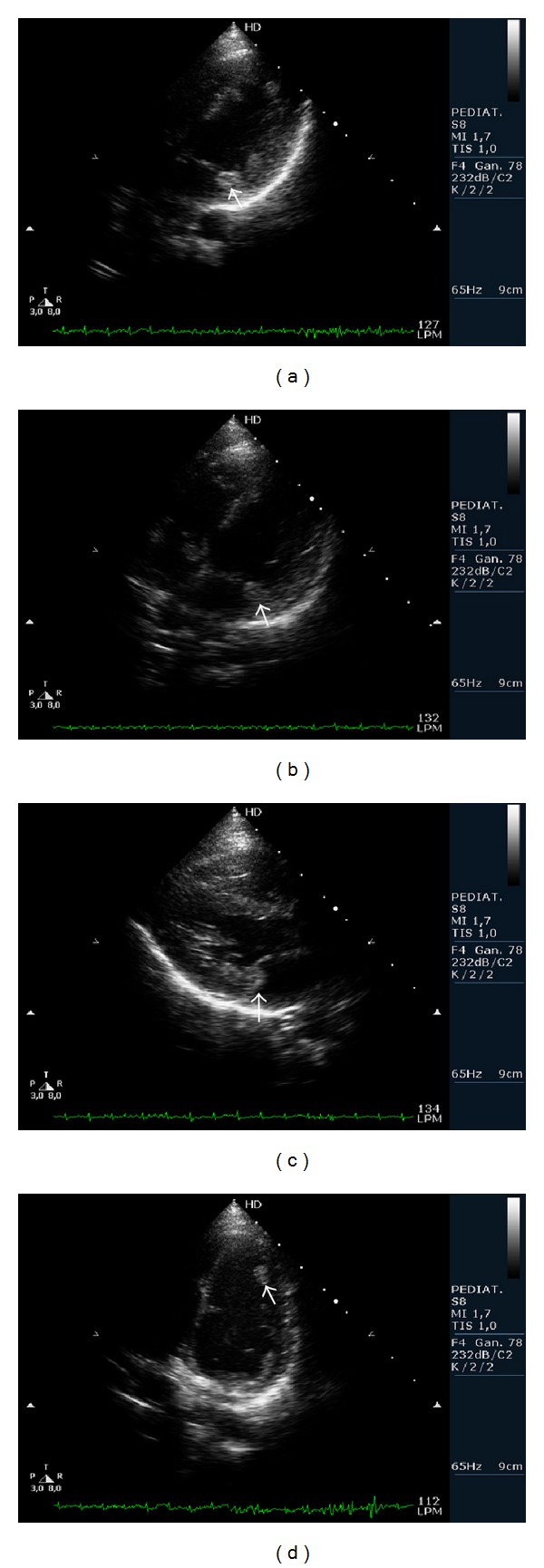
Royalty rhabdomyomas after 28-month followup. Note the disappearance of intramural rhabdomyomas and persistence of rhabdomyoma mainly located in the anterior leaflet of the mitral valve. (a) Four-chamber view with intracavitary mass in mitral valve (arrow). Disappearance of intraparietal mass in lateral wall of left atrium viewed in Figure 1(b). (b) Four-chamber doppler-colour view of intracavitary mass in tricuspid valve smaller than before, one intracavitary mass in mitral valve and disappearance of intraparietal mass in lateral wall of left atrium (arrow). (c) Longitudinal long axis of left ventricule with intracavitary mass on mitral valve (arrow). (d) Four-chamber view of intraparietal tumour in left ventricule apex smaller than before (arrow).

Twenty-eight months after followup, the girl remained asymptomatic with propranolol 0,5 mg/mL, with only small skin lesions, suggestive of cutaneous fibroids, appearing. There were no new episodes of supraventricular tachycardia, and neurological development remained normal. Echocardiographies showed progressive intramural tumors regression that almost disappeared, while the intracavitary remained with a similar size (Figure 2).

## 3. Discussion

Tuberous sclerosis is a neurocutaneous syndrome with autosomal dominant inheritance and has a reported incidence of 1 : 6.000. Formerly recognised by the clinical trial of epilepsy, mental retardation, and facial angiofibromatosis, it is appreciated that almost any organ of the body may be affected. The most common cardiac manifestation of the disease is the cardiac rhabdomyoma, which is thought to occur in at least 60% of children with tuberous sclerosis. A review of all papers on cardiac rhabdomyomas published up to 1990 is available [2]. At least 51% of tumours were associated with tuberous sclerosis and this rose to 86% if all patients with a possible diagnosis of tuberous sclerosis were included [2]. These benign tumours seem to originate from embryonic myocytes, representing hamartomas. The involution may be related to the inability of the tumours to divide while the heart chambers grow. Rhabdomyomas are usually highly reflective tumours, arising from the myocardium, but may be intramural, complicating the diagnosis. If a patient with tuberous sclerosis has multiple cardiac tumours on echocardiography, they are generally considered to be cardiac rhabdomyomas [3]. Furthermore, in view of the high frequency of rhabdomyomas in infants with tuberous sclerosis, echocardiography has been proposed by some as a diagnostic tool when tuberous sclerosis is suspected in infants, even in the absence of other clinical signs, which is common at this age [1].

Cardiac rhabdomyomas have been reported to regress spontaneously in the first years of life, although the regression mechanism is not yet well understood. Recently, in a series of 154 patients with tuberous sclerosis, partial regression of the cardiac rhabdomyomas was reported in 50% of cases and complete resolution was showed in 18% of cases [4]. An earlier report found regression rates of 60% in preadolescent tuberous sclerosis patients and 18% in adult tuberous sclerosis patients [4, 5]. Our patient has the distinction of being one of the few published cases with intramural rhabdomyomas regression with persistent intracavitary rhabdomyomas, which remained in similar size in the two-year followup [6]. The explanation for the regression of only intramural rhabdomyomas is currently unknown, but it does weigh on the possible involvement of embryogenesis, which may be different in each type of rhabdomyoma.

Cardiac rhabdomyomas are typically asymptomatic and are therefore usually not operated upon unless they are obstructive, cause heart failure, or are complicated with severe intractable arrhythmias [7]. Also, they can be difficult to remove completely, because they are often located in the deep myocardium. No embolic events have been reported and there is no need for oral anticoagulation in the absence of a specific indication (e.g., atrial fibrillation). Although there are no consistent guidelines, cardiac monitoring may be proposed for all tuberous sclerosis patients with rhabdomyomas, with serial annual or biannual echocardiograms to detect haemodynamic compromise and annual Holter monitoring to detect severe arrhythmias, even if most patients are usually free from cardiac symptoms. When symptoms are present, they are generally related to the size of the tumours and their location. In our patient echocardiogram after twenty-eight months of followup showed partial regression of rhabdomyomas, as explained previously, and Holter monitoring showed sinusal rhythm with WPW syndrome with 85 beats/min.

There have also been a few isolated reports of Wolff-Parkinson-White syndrome occurring in association with tuberous sclerosis, with and without rhabdomyomas, and its prevalence is not well known. The aetiology of Wolff-Parkinson-White syndrome in tuberous sclerosis has not been explained. It has been known for some time that some of the cells in the cardiac rhabdomyomas found in patients with tuberous sclerosis are structurally identical to normal Purkinje cells, so it has been presumed that rhabdomyomatous tissue traversing the atrioventricular junction acts as the accessory pathway bypassing the atrioventricular node. This occurs more often in males and almost exclusively in association with cardiac rhabdomyomas. It presents early in life, often on day 1, and is usually associated with symptomatic supraventricular tachycardia. It responds well to medical treatment and a high proportion of cases will resolve over time, as it occurs in the present case [8] with 1 mg/8 hours of oral propranolol. To our knowledge this is the only case so far published, bringing together in one case the presence of tuberous sclerosis associated with WPW syndrome and cardiac rhabdomyomas, which present unusual regression with persistent intracardiac rhabdomyomas and disappearance of those intramurals.
